# The risk factors of concomitant intraperitoneal and retroperitoneal hemorrhage in the patients with blunt abdominal trauma

**DOI:** 10.1186/1749-7922-10-4

**Published:** 2015-01-27

**Authors:** Chun-Yi Wu, Shang-Ju Yang, Chih-Yuan Fu, Chien-Hung Liao, Shih-Ching Kang, Yu-Pao Hsu, Being-Chuan Lin, Kuo-Ching Yuan, Shang-Yu Wang

**Affiliations:** Department of Trauma and Emergency Surgery, Chang Gung Memorial Hospital, Chang Gung University, 5, Fu-Hsing Street, Kwei Shan Township, Taoyuan, Taiwan

**Keywords:** Pelvic fracture, Laparotomy, Transcatheter arterial embolization, Tile B_1_

## Abstract

**Introduction:**

Intraperitoneal and retroperitoneal hemorrhages may occur simultaneously in blunt abdominal trauma (BAT) patients. These patients undergo emergency laparotomies because of concomitant unstable hemodynamics and positive sonographic examination results. However, if the associated retroperitoneal hemorrhage is found intraoperatively and cannot be controlled surgically, then the patients require post-laparotomy transcatheter arterial embolization (TAE). In the current study, we attempted to determine the risk factors for post-laparotomy TAE.

**Materials and methods:**

Patients with concomitant BAT and unstable hemodynamic were retrospectively analyzed. The characteristics of the patients who underwent laparotomy or who required post-laparotomy TAE were investigated and compared. The Tile classification system was used to evaluate the pelvic fracture patterns.

**Results:**

Seventy-four patients were enrolled in the study. Fifty-nine (79.7%) patients underwent laparotomy to treat intra-abdominal hemorrhage, and fifteen (20.3%) patients underwent additional post-laparotomy TAE because of concomitant retroperitoneal hemorrhage. Pelvic fracture was present in 80.0% of the post-laparotomy TAE patients. This percentage was significantly greater than that of the laparotomy only patients (80.0% vs. 30.5%, *p* < 0.001). Furthermore, 30 patients (40.5%, 30/74) had concomitant pelvic fracture diagnoses. Of these patients, eighteen (60%, 18/30) underwent laparotomy only, while the other twelve patients (40%, 12/30) required post-laparotomy TAE. Compared with the patients who underwent laparotomy only, more patients with Tile B_1_-type pelvic fractures (58.3% vs. 11.1%, *p* = 0.013) required post-laparotomy TAE.

**Conclusion:**

Regarding BAT patient management, the likelihood of post-laparotomy TAE should be considered in patients with concomitant pelvic fractures. Furthermore, more attention should be directed toward patients with Tile B_1_-type pelvic fractures because of the specific fracture pattern and impaction force.

## Introduction

Intraperitoneal and retroperitoneal hemorrhages may occur simultaneously in patients with concomitant blunt abdominal trauma (BAT) and unstable hemodynamics [1–3]. Additionally, BAT management principles have evolved to a non-operative management approach because of advancements in treatment concepts and improvements in diagnostic modalities [4, 5]. however, laparotomies are still necessary in some situations. In contrast, transcatheter arterial embolization (TAE) serves as an effective surgery alternative for retroperitoneal hemorrhage management [6–8]. Therefore, in addition to aggressive resuscitation, either a laparotomy or a TAE is usually needed for the hemostasis management of these patients. Furthermore, some complicated cases require both procedures; however, only limited information is available for hemorrhage origin assessment when only clinical presentation and primary tools are used for the evaluations. Although sonographic examination is utilized in emergency departments (ED) worldwide, it can only detect intraperitoneal hemorrhage and has limitations regarding retroperitoneal hemorrhage surveillence [9–11]. Therefore, it is difficult to identify patients with concomitant intraperitoneal and retroperitoneal hemorrhages during primary evaluations over a short time period.

Patients usually undergo emergency laparotomies because of concomitant unstable hemodynamics and positive sonographic examination results [9, 10, 12]. However, if associated retroperitoneal hemorrhages are found intraoperatively and cannot be controlled surgically, then post-laparotomy TAE is required. The preparation of an angioembolization suite and the gathering of personnel are usually time-consuming, which may delay definitive hemostasis. Furthermore, transporting these patients between the operation and angiographic rooms is risky under such critical conditions. Therefore, the early identification of these patients is important to ensure that subsequent treatments can be initiated in a timely manner. Additionally, more information may be needed for physicians to predict retroperitoneal hemorrhages and further hemostatic procedure requirements.

In this study, we describe a retrospective observation of the management of patients with concomitant BAT and unstable hemodynamics. The different clinical course characteristics were delineated and compared, and the patients who underwent post-laparotomy TAE were investigated and analyzed. We attempted to determine the risk factors that indicated post-laparotomy TAE and analyzed the critical decision-making processes that occurred when only limited information was available.

## Materials and methods

**Figure 1:**
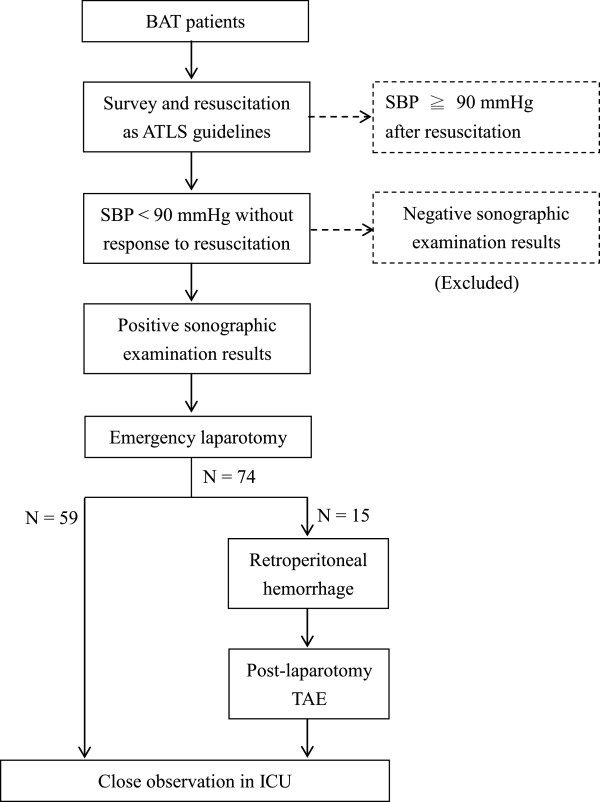
**The established BAT patient protocol used at our institution.**

From May 2008 to October 2013, patients with concomitant BAT and unstable hemodynamic were retrospectively analyzed. They were evaluated and treated according to our established algorithm, which is based on the Advanced Trauma Life Support (ATLS) guidelines [13] (Figure 1). The Pelvic X-ray was routinely used as an adjunct to the primary survey in these patients. Unstable hemodynamics were defined as having systolic blood pressure (SBP) < 90 mmHg, without response to 2000 ml of fluid resuscitation. Patients who died in the ED without undergoing other evaluations were not enrolled in the current study. The pelvic circumferential compression device (PCCD) was applied in the ED while the pelvic fracture diagnosed. Then the patients underwent emergency laparotomy and had positive sonographic examination results, which indicated intra-abdominal hemorrhage. Post-laparotomy TAE was indicated for the patients with retroperitoneal hemorrhages that were found intraoperatively. They were moved to an angiographic room after a damage-control laparotomy after intraperitoneal/retroperitoneal packing directly without additional computed tomographic (CT) scan. These patients were taken to the intensive care unit (ICU) for further observation after these hemostasis procedures. In our institution, the operating and angiography rooms were available 24 hours per day, and a TAE could be performed within one hour.

In this study, the characteristics of the patients who required post-laparotomy TAEs were noted and investigated. Patient demographics, Revised Trauma Scores (RTSs), Injury Severity Scores (ISSs) and number of blood transfusions in the ED were routinely recorded. The Tile classification system was used to evaluate the pelvic fracture patterns [14, 15]. The pelvic ring has a stable fracture with intact sacroiliac complex in type A injuries. The type B and C injuries were classified as unstable pelvic fracture (B_1_: external rotational unstable, B_2_: internal rotational unstable, C: both rotational and vertical unstable). The patients who underwent laparotomy only or laparotomy plus post-laparotomy TAE were compared.

All data are presented as patient percentages or means with standard deviations. The numerical data were compared using the Wilcoxon two-sample exact test. Nominal data were compared using Fisher’s exact test. Multivariate logistic regression analysis was performed to determine the independent risk factors related to the requirement for post-laparotomy TAE. All statistical analyses were performed using the SPSS computer software package (version 13.0, Chicago, IL, USA). A value of *p* < 0.05 was considered to be significant.

## Results

During the 68-month study period, 3,871 patients with BAT diagnoses were sent to our ED. Thirty-nine patients died in the ED without undergoing further evaluations and were excluded. A total of 74 patients with concomitant BAT and unstable hemodynamics without response to 2000 ml of fluid resuscitation were enrolled in this study. The mean patient age was 42.0 years (48 [64.7%] males and 26 [35.3%] females).

The various clinical course distributions for the patients are shown in Figure 1. Fifty-nine (79.7%) patients underwent laparotomy only to treat intra-abdominal hemorrhage, and fifteen (20.3%) patients underwent post-laparotomy TAE because of concomitant retroperitoneal hemorrhage. Table 1 compares the patients who underwent laparotomy only with patients who required post-laparotomy TAE. Pelvic fracture was present in 80.0% of the patients who required post-laparotomy TAE, which was significantly greater than the percentage of the patients who underwent laparotomy only (80.0% vs. 30.5%, *p* < 0.001). These patients had significantly higher ISSs (24.6 ± 24.1 vs. 19.5 ± 20.7, *p* = 0.004), lower RTSs (4.819 ± 1.335 vs. 6.007 ± 0.772, *p* = 0.011) and a greater amount of transfused blood (1356.3 ± 977.6 ml vs. 887.3 ± 934.9 ml, *p* = 0.028) (Table 1). Multivariate logistic regression analyses revealed that the presence of a pelvic fracture (odds ratio [OR] = 3.4, *p* = 0.018) and an ISS ≧ 16 (OR = 2.2, *p* = 0.048) were two significant predictive factors for patients requiring post-laparotomy TAE (Table 2).

**Table 1:** **Comparisons between the patients who underwent laparotomy only and the patients who required post-laparotomy TAE**

Variables	Laparotomy only (N = 59)	Laparotomy → TAE (N = 15)	***p*** -value
Age	42.3 ± 24.2	40.6 ± 23.1	0.916^#^
Gender (N)			0.367^$^
Female	19 (32.2%)	7 (46.7%)	
Male	40 (67.8%)	8 (53.3%)	
ISS	19.5 ± 20.7	24.6 ± 24.1	0.004^#^
RTS	6.007 ± 0.772	4.819 ± 1.335	0.011^#^
Blood transfusion (ml)	887.3 ± 934.9	1356.3 ± 977.6	0.028^#^
Pelvic fracture (N)			< 0.001^$^
Yes	18 (30.5%)	12 (80.0%)	
No	41 (69.5%)	3 (20.0%)	
Pelvis stability			< 0.001^$^
Stable (tile A)	55 (93.2%)	6 (40.0%)	
Unstable (tile B/C)	4 (6.8%)	9 (60.0%)	

The variables are expressed as means ± SD.

^#^Wilcoxon two-sample exact test, ^$^Fisher’s exact test.

**Table 2:** **The factors independently associated with post-laparotomy TAE in the overall patient population**

Variable	Odds ratio (95% CI)	***p*** -value ^∫^
Pelvic fracture	3.4 (2.2 ~ 11.4)	0.018
Blood transfusion ≧ 1500 ml	8.7 (0.7 ~ 15.2)	0.302
RTS < 5.5	5.2 (0.1 ~ 13.4)	0.272
ISS ≧ 16	2.2 (1.6 ~ 9.5)	0.048

^∫^Multivariate logistic regression.

In the current study, there were 30 concomitant pelvic fracture diagnoses (40.5%, 30/74). Of these patients, eighteen (60%, 18/30) underwent laparotomy only, while the other twelve patients (40%, 12/30) required post-laparotomy TAE. Compared with the laparotomy-only patients, the post-laparotomy TAE patients required more transfused blood (1542.8 ± 1022.5 ml vs. 914.5 ± 425.9 ml, *p* < 0.001). Additionally, there were more patients with type B_1_ pelvic fractures (58.3% vs. 11.1%, *p* = 0.013) or unstable pelvic fractures (75.0% vs. 22.2%, *p* = 0.008) among these patients. Furthermore, the post-laparotomy TAE patients also demonstrated significantly higher ISSs (26.3 ± 14.1 vs. 22.5 ± 20.7, *p* = 0.004) and lower RTSs (4.115 ± 2.431 vs. 5.981 ± 3.212, *p* = 0.011) than the other patients (Table 3). Table 4 shows that a type B_1_ pelvic fracture (OR = 6.4, *p* = 0.002) and an ISS > 16 (OR = 5.9, *p* = 0.002) were two independent risk factors for post-TAE laparotomy.

**Table 3:** **Comparisons between the pelvic fracture patients who underwent laparotomy only and the patients who required post-laparotomy TAE**

Variables	Laparotomy only (N = 18)	Laparotomy → TAE (N = 12)	***p*** -value
Age	41.8 ± 16.7	43.0 ± 22.6	0.854^#^
Gender (N)			1.000^$^
Female	5 (27.8%)	4 (33.3%)	
Male	13 (72.2%)	8 (66.7%)	
ISS	22.5 ± 20.7	26.3 ± 14.1	0.004^#^
RTS	5.981 ± 3.212	4.115 ± 2.431	0.011^#^
Blood transfusion (ml)	914.5 ± 425.9	1542.8 ± 1022.5	< 0.001^#^
Fracture pattern (N)			0.013^$^
Tile B_1_	2 (11.1%)	7 (58.3%)	
Non-Tile B_1_ (A + B_2_ + C)	16 (88.9%)	5 (41.7%)	
Pelvis stability			0.008^$^
Stable (tile A)	14 (77.8%)	3 (25.0%)	
Unstable (tile B/C)	4 (22.2%)	9 (75.0%)	

Variables are expressed as means ± SD.

^#^Wilcoxon two-sample exact test, ^$^Fisher’s exact test.

**Table 4:** **The factors independently associated with post-laparotomy TAE in pelvic fracture patients**

Variable	Odds ratio (95% CI)	***p*** -value ^∫^
Tile B_1_ pelvic fracture	6.4 (4.0 ~ 15.1)	0.002
Blood transfusion ≧ 1500 ml	2.9 (0.1 ~ 4.6)	0.519
RTS < 5.5	3.3 (0.4 ~ 5.1)	0.238
ISS ≧ 16	5.5 (3.6 ~ 14.2)	0.001

^∫^Multivariate logistic regression.

In current study, there were three patients without pelvic fractures who required post-laparotomy TAE. Their angiographic examinations revealed that all of them had active lumbar arterial hemorrhage.

## Discussion

Blunt abdominal trauma may result in intraperitoneal or retroperitoneal hemorrhages, which are both potentially life-threatening and require organized rapid evaluation and treatment [1–3]. An emergency laparotomy is usually indicated for an intraperitoneal hemorrhage that presents as intra-abdominal free fluid during sonography. In the management of the associated retroperitoneal hemorrhage, the PCCD and even the extraperitoneal packing are required to decrease the retroperitoneal hemorrhage before the laparotomy. Then the further TAE could be performed for the definitive hemostasis. However, in these patients, it is difficult to consider the requirement of TAE until persistent expansion of a retroperitoneal hematoma was detected intraoperatively. At that time, the TAE preparation would be initiated. After a damage-control laparotomy, with intraperitoneal/retroperitoneal packing, the patients would be sent to an angiographic room. Therefore, they would remain in critical condition with an ongoing hemorrhage while waiting for an angioembolization suite to be prepared and for personnel to be gathered.

In the current study, 74 patients underwent emergency laparotomy because of concomitant unstable hemodynamics and positive sonographic examination results. Retroperitoneal hemorrhage was found intraoperatively in 15 of these patients (20.3%); thus, they underwent post-laparotomy TAE. The percentage of these patients with concomitant pelvic fracture was significantly greater than that of the patients who underwent laparotomy only (80.0% vs. 30.5%, *p* < 0.001). Furthermore, multivariate logistic regression analyses revealed that the presence of a pelvic fracture was a significant factor in predicting the need for post-laparotomy TAE in such patients (OR = 3.4, *p* = 0.018) (Table 2). Pelvic fractures usually stem from high-kinetic-energy blunt trauma and can result in retroperitoneal hemorrhage. Associated hemodynamic instability was reported in 5–20% of these patients, and the subsequent reported mortality rate was 18–40% [16–20]. Therefore, in addition to intraperitoneal hemorrhage, an associated retroperitoneal hemorrhage should also be considered when managing patients with BAT and unstable hemodynamics with concomitant pelvic fractures.

Previous reports have proposed that unstable pelvic fractures indicate major ligamentous disruptions that are often associated with life threatening arterial bleeding [14, 15, 21]. Additionally, pelvic fracture patterns were classified into three main groups according to the integrity of the posterior sacroiliac complex by Tile [14, 15]. The focused analysis of pelvic fracture patients in the current study revealed that the patients who required post-laparotomy TAE had a significantly greater percentage of unstable pelvises (75.0% vs. 22.2%, *p* = 0.008) and type B_1_ (external rotational unstable) pelvic fractures (58.3% vs. 11.1%, *p* = 0.013) compared with the patients who underwent laparotomy only (Table 3). Furthermore, multiple logistic regression analyses in the current study also revealed that type B_1_ pelvic fractures were an independent risk factor for concomitant intraperitoneal and retroperitoneal hemorrhages, with approximately an eight-fold increased risk of requiring post-laparotomy TAE (OR = 6.4, *p* = 0.002) (Table 4).

**Figure 2:**
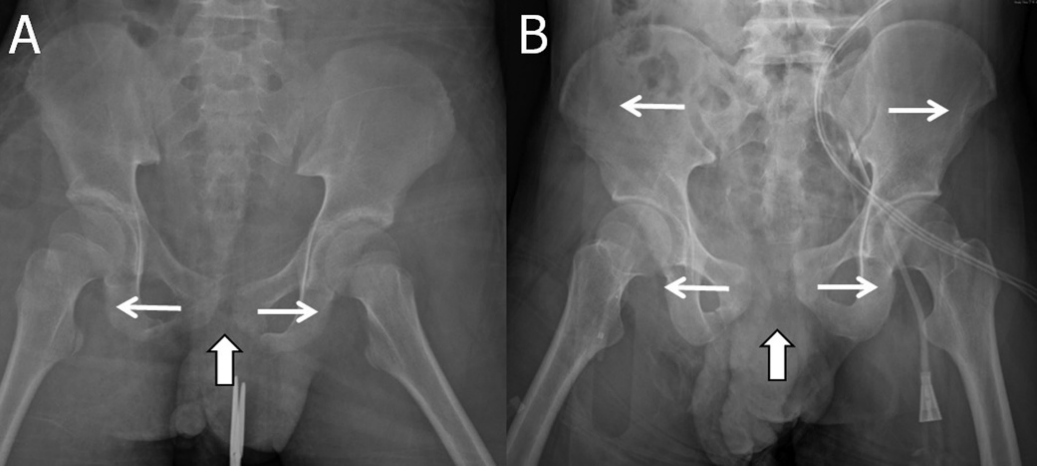
**Plain external rotational type pelvic fracture films.** The large arrows indicate the disruptive force direction and side of the impact, and the small arrows indicate the splaying of the pubic symphysis **(A)** and further external iliac wing rotation **(B)**.

Unlike other pelvic fractures, type B_1_ pelvic fractures create complete anterior pelvis diastases and partial or complete posterior pelvis diastases (Figure 2) [15, 18, 22]. The tensile and shearing force of such injuries are generally from the middle and front. They tend to open the anterior pelvis, which is thinner than the iliac bone, over the lateral side by pubic ramus fracture or pubic symphysis ligaments rupture mechanisms (Figure 2A). Furthermore, additional external rotational forces may result in splaying of the anterior pelvis and external rotation of the iliac wings, which provide circumferential protection of the intra-abdominal organs (Figure 2B). Therefore, it is reasonable that such impaction, with relative weak protection, may be associated with a greater risk of intraperitoneal injury. Additionally, it was reported that the external rotational pelvic fracture involves greater vessel disruption and more hemorrhaging than other injury mechanisms [22]. Our previous report also indicated its association with bilateral sacroiliac joint injuries and further bilateral arterial injuries [23]. According to the above theories, concomitant intraperitoneal and retroperitoneal hemorrhages may occur in type B_1_ pelvic fracture patients. In managing patients with unstable hemodynamics and positive sonographic examinations, associated retroperitoneal hemorrhages should be suspected in patients with concomitant type B_1_ pelvic fractures.

Another concern is in evaluating other associated injuries. The ISS has been used to evaluate the severity of other associated injuries [24, 25]. This trauma scoring system is a tool that can assist with the triage, prognosis prediction and resource allocation of ED trauma patients. In the current study, the post-laparotomy TAE patients also had significantly higher ISSs than the patients who underwent laparotomy only. Multiple logistic regression analysis also revealed that a higher ISS (≥16) was independently associated with an increased risk for post-laparotomy TAE. Although the precise ISS was not available in the ED, the current study results indicate that other associated injuries may increase the risk of subsequent deterioration requiring additional procedures.

The early identification of post-laparotomy TAE patients might shorten the waiting time for TAE. Furthermore, the risk that occurs while patients wait and during transportation could also be reduced. Pelvic fractures can easily be diagnosed on a plain pelvis film; thus, type B_1_ pelvic fractures could also be identified accordingly. This fact revealed that concomitant retroperitoneal hemorrhages and the necessity of additional hemostatic procedures for unstable patients could be surmised with primary tool evaluations (e.g., sonographic examinations and plain pelvis films). To save additional time, one suggestion is to simultaneously prepare for TAE while performing laparotomy in type B_1_ pelvic fracture patients. This process may diminish the increased risk of injuries associated with concomitant retroperitoneal hemorrhages, which may require post-laparotomy TAE.

The limitations of this study are its retrospective nature and small number of examined cases. Our conclusions may be limited by a possible selection bias. Additionally, the role of CT scan was not discussed in the current study. We agree that CT scan could augment the detection of soft tissue injuries and active arterial bleeding; however, CT scans are not recommended for most patients with unstable hemodynamics [13]. Further studies with larger sample sizes and prospective designs are needed to establish precise treatment plan algorithms in the ED.

## Conclusion

In BAT patient management, the likelihood of post-laparotomy TAE should be considered in concomitant pelvic fracture patients. Furthermore, more attention should be directed toward patients with Tile B_1_-type pelvic fractures because of its specific fracture pattern and impaction force.

## Authors’ information

Chun-Yi Wu and Shang-Ju Yang are first author.
